# Supplementary material for: Impact of Accessory Corpus Luteum Induced by Gonadotropin-Releasing Hormone or Human Chorionic Gonadotropin on Pregnancy Rates of Dairy Cattle following Embryo Transfer: A META-Analysis
Source: Vet Sci. 2023 Apr 23;10(5):309. doi: 10.3390/vetsci10050309 (PMC10224191; doi:10.3390/vetsci10050309)
Supplement: Supplementary file 1 [file vetsci-10-00309-s001.zip › vetsci-2266075-supplementary.pdf]

Supplementary Figure S1. Treatment with hCG or GnRH on pregnancy rates of heat-stressed dairy cows.

	Experimental		Control			Risk Ratio
**Study or Subgroup**	**Events**	**Total**	**Events**	**Total**	**Weight**	**M-H, Fixed, 95% CI**
Block 2003	22	85	7	63	6.0%	2.33 [1.06, 5.11]
Franco 2006	8	45	7	42	5.4%	1.07 [0.42, 2.68]
Pinto 2015	54	175	19	92	18.4%	1.49 [0.95, 2.36]
Vasconcelo 2011 a	51	124	48	126	35.3%	1.08 [0.79, 1.47]
Vasconcelo 2011 b	65	122	48	126	35.0%	1.40 [1.06, 1.85]
**Total (95% CI)**		**551**		**449**	**100.0%**	**1.34 [1.12, 1.61]**
Total events	200		129			

Heterogeneity: $Chi^2 = 4.36$, df = 4 (P = 0.36); $I^2 = 8\%$

Test for overall effect: Z = 3.17 (P = 0.002)

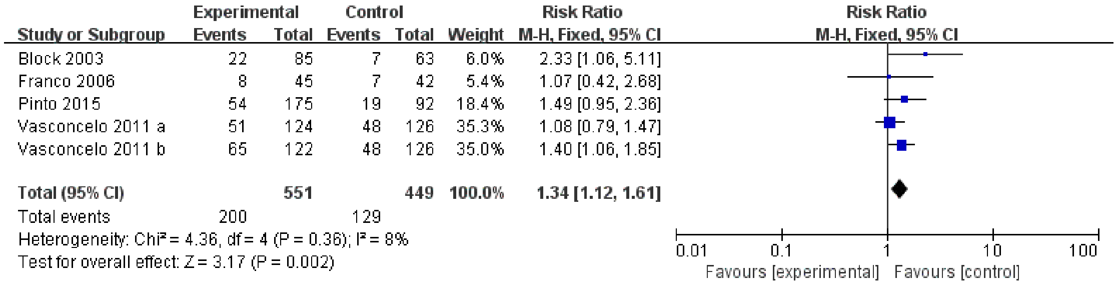

Supplementary Table 1. Interaction test

Variables	Parity	Treatment type	Dosage	Treatment time
Parity	--	0.45	0.62	0.82
Treatment type	0.45	--	0.87	0.55
Dosage	0.62	0.87	--	0.16
Treatment time	0.82	0.55	0.16	--
